# miRCat2: accurate prediction of plant and animal microRNAs from next-generation sequencing datasets

**DOI:** 10.1093/bioinformatics/btx210

**Published:** 2017-04-12

**Authors:** Claudia Paicu, Irina Mohorianu, Matthew Stocks, Ping Xu, Aurore Coince, Martina Billmeier, Tamas Dalmay, Vincent Moulton, Simon Moxon

**Affiliations:** 1 The Earlham Institute, Norwich Research Park, Norwich, UK; 2 School of Computing Sciences, University of East Anglia, Norwich Research Park, Norwich, UK; 3 School of Biological Sciences, University of East Anglia, Norwich Research Park, Norwich, UK

## Abstract

**Motivation:**

MicroRNAs are a class of ∼21–22 nt small RNAs which are excised from a stable hairpin-like secondary structure. They have important gene regulatory functions and are involved in many pathways including developmental timing, organogenesis and development in eukaryotes. There are several computational tools for miRNA detection from next-generation sequencing datasets. However, many of these tools suffer from high false positive and false negative rates. Here we present a novel miRNA prediction algorithm, miRCat2. miRCat2 incorporates a new entropy-based approach to detect miRNA loci, which is designed to cope with the high sequencing depth of current next-generation sequencing datasets. It has a user-friendly interface and produces graphical representations of the hairpin structure and plots depicting the alignment of sequences on the secondary structure.

**Results:**

We test miRCat2 on a number of animal and plant datasets and present a comparative analysis with miRCat, miRDeep2, miRPlant and miReap. We also use mutants in the miRNA biogenesis pathway to evaluate the predictions of these tools. Results indicate that miRCat2 has an improved accuracy compared with other methods tested. Moreover, miRCat2 predicts several new miRNAs that are differentially expressed in wild-type versus mutants in the miRNA biogenesis pathway.

**Availability and Implementation:**

miRCat2 is part of the UEA small RNA Workbench and is freely available from http://srna-workbench.cmp.uea.ac.uk/.

**Supplementary information:**

Supplementary data are available at *Bioinformatics* online.

## 1 Introduction

MicroRNAs (miRNAs) are a class of small non-coding RNAs (sRNAs) that are excised from a hairpin-like secondary structure of a primary transcript (Bartel, 2004; Kim, 2005). They are present and functional in metazoa and in some viruses; their mode of action consists of the downregulation of the target gene(s) through post-transcriptional silencing (Bartel, 2004; Chen, 2005; Kim, 2005). The identification and characterization of miRNAs, which are ∼21–22 nt in length, has developed as a major research topic due to their important role in gene regulation and influence on pathways such as hematopoiesis, apoptosis, cell proliferation and tumorgenesis (Cheng *et al.*, 2005; Iorio *et al.*, 2005; Esquela-Kerscher and Slack, 2006; Jones-Rhoades *et al.*, 2006; Lu *et al.*, 2008; Pérez-Quintero *et al.*, 2010).

### 1.1 miRNA biogenesis and function

In animals, miRNA genes are transcribed by RNA polymerase II to generate long capped and polyadenylated transcripts (termed pri-miRNAs) (Cai *et al.*, 2004; Lee *et al.*, 2004; Kim, 2005; Xie *et al.*, 2015). The Drosha protein recognizes the hairpin structure of the pri-miRNA and initiates the first processing step (cropping) (Lee *et al.*, 2003; Denli *et al.*, 2004; Gregory *et al.*, 2004; Han *et al.*, 2004; Kim, 2005; Zeng *et al.*, 2005). The product of this nuclear processing step is a ∼70 nt precursor (pre-miRNA), which folds into a short stem-loop structure with a ∼2 nt 3′ overhang (Kim, 2005). A nuclear export factor (Exportin-5) recognizes this structure as a signature motif and exports it from the nucleus to the cytoplasm (Yi *et al.*, 2003; Bohnsack *et al.*, 2004; Bartel, 2004; Lund *et al.*, 2004; Kim, 2005). Here, a Dicer protein removes the loop-region and gives rise to the miRNA duplex (process known as ‘dicing’) (Bernstein *et al.*, 2001; Grishok *et al.*, 2001; Hutvágner *et al.*, 2001; Ketting *et al.*, 2001; Bartel, 2004; Kim, 2005; Ha and Kim, 2014). The duplex is then separated and usually one strand is selected as the mature miRNA, whereas the other strand may be degraded; in some cases both 3′ and 5′ miRNAs are stable and functional (Khvorova *et al.*, 2003; Ha and Kim, 2014).

The biogenesis of miRNAs in plants is similar to that of animals, with some differences. Although in animals the length and structure of the pre-miRNA hairpin is fairly consistent, in plants it is longer and much more variable (100–300 nt) (Cuperus *et al.*, 2011). A DICER-LIKE1 (DCL1) enzyme excises the miRNA duplex from the pri-miRNA, in the nucleus (Park *et al.*, 2002; Reinhart *et al.*, 2002; Papp *et al.*, 2003; Kurihara and Watanabe, 2004; Xie *et al.*, 2004), then the small RNA methyltransferase hua enhancer1 (HEN1) adds a methyl group to the 3′ end to stabilize it (Yu *et al.*, 2005; Xie *et al.*, 2015). The duplex is then transported from the nucleus to the cytoplasm by hasty (HST), a homolog of exportin 5 (Chen, 2005; Xie *et al.*, 2015). The duplex is then separated in the cytoplasm, giving rise to the mature miRNAs (Chen, 2005; Xie *et al.*, 2015).

The mature miRNA is incorporated into the RNA-induced silencing complex (Bartel, 2004; Eamens *et al.*, 2009; Wu *et al.*, 2009; Fabian and Sonenberg, 2012), where it is bound by AGO proteins and guides the complex to complementary messenger RNA sequences (usually within the 3′ UTR, in animals, and within the coding region, in plants) (Bartel, 2009; Bazzini *et al.*, 2012; Djuranovic *et al.*, 2012; Ameres and Zamore, 2013). miRNAs can regulate critical cellular and developmental processes (Cheng *et al.*, 2005; Iorio *et al.*, 2005; Esquela-Kerscher and Slack, 2006; Lu *et al.*, 2008; Ameres and Zamore, 2013). In plants, miRNAs are also involved in diverse responses to stresses such as drought, salt, cold, oxidative, nutrient deficiency as well as biotic stresses (Jones-Rhoades *et al.*, 2006; Pérez-Quintero *et al.*, 2010; Xie *et al.*, 2015).

### 1.2 Computational detection of miRNAs

Over the last decade, various computational tools have been developed for identifying miRNAs from next-generation sequencing (NGS) datasets, using features of the miRNA biogenesis. Some of the more commonly used tools, in temporal order of appearance, are: miRDeep (Friedländer *et al.*, 2008), miRCat (Moxon *et al.*, 2008), miReap (http://mireap.sourceforge.net/), MIReNA (Mathelier and Carbone, 2010), miRAnalyzer (Hackenberg *et al.*, 2009), miRDeep-P (Yang and Li, 2011), miRDeep2 (Friedländer *et al.*, 2012), MaturePred (Xuan *et al.*, 2011), miRDeep* (An *et al.*, 2013), miRAuto (Lee *et al.*, 2013), miRPlant (An *et al.*, 2014), miR-PREFeR (Lei and Sun, 2014), Mirinho (Higashi *et al.*, 2015) and miRA (Evers *et al.*, 2015). Many of these approaches, including the miRCat tool, suffer from high false positive and false negative rates and also lack of consistency across species (Li *et al.*, 2012; Williamson *et al.*, 2012; Kang and Friedländer, 2015).

The miRCat algorithm groups reads on proximity on the reference genome. It then selects one candidate from each locus and computes discriminative features on their secondary structure, to classify them as miRNAs. miRCat was introduced when NGS sequencing depth was typically orders of magnitude smaller compared to current NGS datasets. The higher sequencing depth strongly influences the grouping approach which can result in high false positive and high negative rates (Mohorianu *et al.*, 2013). Sequencing depth is also problematic for many of the tools mentioned above, for similar reasons (Tucker *et al.*, 2009; Baker, 2010).

To overcome this, we have developed miRCat2, a new miRNA prediction tool, applicable on both animal and plant datasets, which incorporates elements of its predecessor miRCat (Moxon *et al.*, 2008), and discriminative features from miRDeep2 (Friedländer *et al.*, 2012). miRCat2 implements a new approach to differentiate miRNA candidates from background sequences, then applies novel filters on the candidate sequence alignments and secondary structure. miRCat2 is part of the UEA small RNA Workbench (Stocks *et al.*, 2012) and it has a user-friendly interface, as well as a command-line option, which allows the integration into bioinformatics workflows. The algorithm is performing well on animal datasets; it also allows the detection of complex structures and even multiple miRNA loci within a single precursor in plants.

To assess the performance of miRCat2, we have compared it to miRCat (Moxon *et al.*, 2008), miRDeep2 (animal data) (Friedländer *et al.*, 2008), miRPlant (plant data) (An *et al.*, 2014) and miReap (http://mireap.sourceforge.net/). We have chosen these tools based on their popularity and on benchmarking results (Li *et al.*, 2012; Williamson *et al.*, 2012; Kang and Friedländer, 2015), which, to our best knowledge, classify them as generally the most advantageous methods. The comparison is performed on a number of animal and plant datasets; we also used mutants which affect the miRNA biogenesis pathway to validate the predictions.

## 2 Methods

### 2.1 Overview

In the Supplementary File S1, Figure S1.1, we present an overview of the workflow for the miRCat2 algorithm. After mapping the reads to the reference genome [using PatMaN (Prüfer *et al.*, 2008), full length, with 0 gaps and 0 mismatches], the algorithm selects reads based on abundance, then filters on read alignment patterns and secondary structure of the putative pre-miRNA hairpin. We now present a detailed description of the algorithm.

#### 2.1.1 Selecting candidates

miRCat2 implements a method of candidate selection designed to deal with high depth datasets. As sequencing depth increases, degradation products may obscure miRNA peaks (see Supplementary File S1, Fig. S1.2). To cope with this, we focus on selecting all the peaks at any given genomic location, while discounting sequences with abundances at or below a background level that we compute from the data. It is known that mature miRNAs and their complementary miRNA* sequence generally have higher abundances than non-miRNAs (Lau *et al.*, 2001). When aligning miRNA reads back to the pre-miRNA locus we see characteristic peaks forming, corresponding to the 5′ and 3′ miRNA sequences (Supplementary File S1, Fig. S1.2A). We can use this information to select a restricted group of sequences as candidates, on which further analysis is performed.

To identify putative miRNA loci based on ‘peaks’ of abundance, we use the following procedure:

- The genome is split into consecutive windows of size *l*_w_ nt, with an overlap of *l*_o_ nt (Mohorianu *et al.*, 2013).
- Each window is split into subwindows of size *l*_sw_ and the mapped reads are assigned to subwindows based on location.
- Each window is compared with a random uniform distribution (RUD) on subwindow abundances, using the entropy-based Kullback–Leibler divergence (KLD) (Kullback and Leibler, 1951; Mohorianu *et al.*, 2011), using: $D_{\text{KL}}(P\|Q)=\sum_{i}\left|\ln\left(\frac{P(i)}{Q(i)}\right)\right|P(i)$, where *i* is the index of the subwindow, *Q* is the RUD and *P* is the abundance distribution on the current window. The probabilities for each subwindow are calculated from the read abundances: $P_{\left(i\right)}=\frac{\sum r_{\text{sw}}}{\sum r_{w}}$, where *r*_sw_ represents the abundance of the reads mapping to the subwindow and *r*_w_ represents the abundance of the reads mapping to the window, after a default offset of 1 has been added to each subwindow, to avoid reads with low expression to be classified as peaks. The probability for the RUD is calculated using the following equation: $Q_{\left(i\right)}=\frac{1}{{\#}_{\text{sw}}}$, where ${\#}_{\text{sw}}$ represents the total number of subwindows contained in a window.
- A KLD score close to 0 indicates a uniform distribution, i.e. no peak is present. If the distribution is an RUD, then it is unlikely that an miRNA is present at the given location and the window is discarded. If the KLD is greater than a threshold (rud_val, empirically determined), then the current window contains at least one peak (the method can also detect multiple peaks). In this case, the subwindow with the highest peak is identified and the most abundant sRNA is selected. The KLD is applied again on a restricted area around this sRNA (plateau_range) to avoid detecting a peak that is actually a plateau (multiple neighboring subwindows that are all highly expressed). If this filter is passed, the sRNA is removed from the distribution and saved as an miRNA candidate for further investigation.
- The KLD is recalculated with the new distribution. If the new KLD is still greater than the threshold, steps (c) to (e) are repeated until we reach an RUD (no more peaks). All removed sRNAs are miRNA candidates and are analyzed using the following steps.

#### 2.1.2 Filtering the sequences

After miRNA candidates are selected, potential false positive predictions are excluded from down-stream analysis using a rule-based approach. First, we discard the sequences that map to the genome more than *repeats* times as high-confidence miRNAs are unlikely to be derived from repetitive regions of the genome (Meyers *et al.*, 2008; Kozomara and Griffiths-Jones, 2014) (user-configurable parameter).

Second, a size class distribution filter is applied, allowing us to focus on reads between 21 and 23 nt, which is the expected miRNA range. To check whether the miRNA candidates are within the range, we compute the KLD on size classes, comparing the sRNA size class distribution (*P*) to an RUD on all size classes (*Q*) (Mohorianu *et al.*, 2013). The sequences contributing to the sRNA size class distribution are all the reads incident to the putative miRNA precursor. If the KLD result is >rud_val, then the size class distribution is different from random. We investigate whether the most abundant size class falls between 21 and 23 nt, otherwise the sRNA locus is discarded. As a small set of annotated miRNAs in miRBase fall outside of this size range, these values are configurable (min_len, max_len).

Third, to check whether the candidates have an miRNA-like alignment of incident reads, we also apply a filter that selects sequences with evidence of precise processing of the pre-miRNA by Drosha (animals) and Dicer (plants and animals) (Bartel, 2004; Chen, 2005; Kim, 2005), i.e. the presence of one or two peaks corresponding to the miRNA/miRNA*. This filtering step ensures that the majority of reads aligned to the miRNA/miRNA* location have a high overlap (are variants of each other), and have the same genomic orientation. The distribution of reads of a genuine miRNA should have a similar shape to that shown in the Supplementary File S1, Figure S1.2A compared to a locus generated from random RNA degradation, Figure S1.2B.

We define a cluster as all sequences that map to the same genomic location, having the start and the end of the mapping position within clear_cut nt of each other. The algorithm for the classification of clusters is presented in the Supplementary File S2. We identify all clusters on the window corresponding to each selected miRNA candidate, *s*; next, to evaluate the existence of a precise excision (e.g. resulting from Drosha and/or Dicer cleavage), we use the following criteria: (i) if the sum of the abundances of all sequences with same start and end positions (±clear_cut nt) as *s* represent clear_cut_percent% of the total abundance of the cluster, then *s* is kept for subsequent analysis; otherwise, it is discarded; (ii) if the sum of the abundances of all sRNAs from adjacent clusters that overlap with *s* with more than clear_cut nt represents less than overlap_percent% of the total abundance of the *s* cluster, then *s* is kept for further analysis; otherwise, it is discarded. 

#### 2.1.3 Using the secondary structure to determine the candidate pre-miRNA

Most methods for miRNA prediction extract a fixed, arbitrary flanking region containing the miRNA candidate and fold it using RNA secondary structure prediction tools (Lorenz *et al.*, 2011) to identify a suitable hairpin-like precursor (Moxon *et al.*, 2008; Friedländer *et al.*, 2012; An *et al.*, 2014). However, this approach is highly dependent on the length of the flanking region; therefore choosing an optimal length is a critical step. To address this, we employ RNALfold (Lorenz *et al.*, 2011), previously used by miR-PREFeR (Lei and Sun, 2014) and miRA (Evers *et al.*, 2015), which folds a large window giving all possible structures contained within that region. To detect the most appropriate secondary structure, we consider a window of max_fold_len nt on each side of the miRNA candidate, ensuring that it is wide enough to capture the pre-miRNA structure.

RNALfold outputs a list containing all possible secondary structures for the selected region, in dot-bracket notation, and their corresponding minimum free energies (MFE). To compare the stability of two subwindows of differing lengths, we calculate the adjusted minimum free energy (aMFE), per 100 nt, for each secondary structure, as follows: $\text{aMFE}=\frac{\text{MFE}}{\text{fold}\_\text{length}}*100$. The secondary structures that contain the miRNA candidate are kept for subsequent filtering which includes the evaluation of the hairpin length; maximum aMFE; and features specific to the hairpin structure (full details of parameters are listed in the Supplementary File S3). If there is more than one subwindow whose secondary structure passes all filters, the one with the lowest aMFE is accepted as the true precursor.

miRCat2 computes a score for the proposed precursor calculated based on the miRDeep2 model, as described in Friedländer *et al.* (2008). The score indicates the strength of the prediction, but it does not influence the output of the method. It could be used as a ranking criteria for the results, a higher score meaning the prediction has a higher probability of being a true miRNA.

### 2.2 Implementation

The miRCat2 algorithm is part of the UEA small RNA Workbench (Stocks *et al.*, 2012) and is written in Java, version 1.8+; for optimal results, we recommend using the latest, stable, Java version. It can run on any operating system (Windows, Linux, Mac OSX). In addition, it can be executed either through the user-friendly interface or from the command line. Two sets of default parameters are provided, one for animals and one for plants, although the user can adjust these parameters. The default parameters were set according to rules generally applicable to the annotated miRNAs from miRBase (Kozomara and Griffiths-Jones, 2014) for each specific Kingdom. A list of all parameters and their default values is presented in the Supplementary File S3.

miRCat2 requires as input a reference genome and a set of sRNA sequencing data (fasta format, non-redundant, with the adaptors trimmed). The files can be processed from fastq to the necessary format using the UEA small RNA Workbench (Stocks *et al.*, 2012). The environment can also be used to map the reads to the reference genome using PatMaN (Prüfer *et al.*, 2008), full length, with 0 gaps and no mismatches. The sequences not mapping to the reference genome are discarded.

**Fig. 1:**
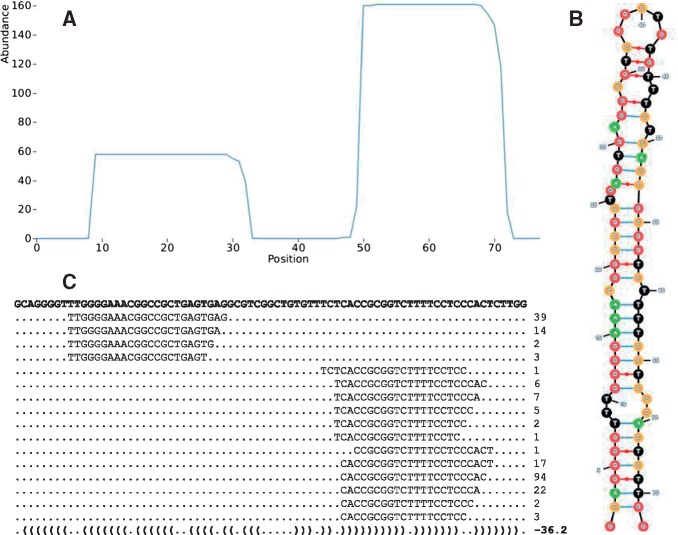
Output of miRCat2 for a predicted sequence corresponding to hsa-mir-2110 (chromosome 10), depicting (**A**) precursor presence plots, (**B**) precursor secondary structure and (**C**) alignment of incident reads. **(A)** On the *x*-axis we represent each position along the miRNA hairpin; on the *y*-axis we represent the point abundance calculated as the algebraic sum of the abundances of incident reads. **(B)** Precursor secondary structure, color-coded for each nucleotide type (A—green, C—orange, G—red, T—black). **(C)** Alignment of incident reads on the precursor; the numbers of the right represent the raw read abundance. The last line presents the secondary structure in dot-bracket notation, together with its MFE

The output of miRCat2 is presented as (i) a PatMaN file, containing the predicted miRNA coordinates; (ii) a csv file, containing additional information about the miRNA*, hairpin and existing miRNA annotations; (iii) a PDF file including, for every predicted miRNA precursor, coverage plots of mapped abundances; and (iv) a text file containing, for every prediction, the read alignments on the precursor (Fig. 1).miRCat2 uses RNALfold from the ViennaRNA package (Lorenz *et al.*, 2011) for detecting the secondary structure, randfold (Bonnet *et al.*, 2004) for calculating the statistical significance of the precursor structure. All dependencies are included in the download package and no extra installation is required. The code can be downloaded from http://srna-workbench.cmp.uea.ac.uk/downloadspage/, where users can also find the documentation and example files.

### 2.3 Fold change computation

To validate miRNA predictions, we estimate fold changes between wild-type and mutants in the miRNA biogenesis pathway. To do this, we consider only the genome mapping reads. To compare datasets with different sequencing depths, we normalize all abundances using the reads per million method (Mortazavi *et al.*, 2008) to the median total count of each experiment (McCormick *et al.*, 2011; Dillies *et al.*, 2013). The method used for calculating the fold changes between wild-type and mutants in the miRNA biogenesis pathway is presented in the Supplementary File S2.

## 3 Materials

### 3.1 Data

To assess the performance of miRCat2, we ran it on multiple organisms and benchmarked the results against other commonly used miRNA detection tools, miRCat (version srna-workbenchV3.2), miRDeep2 (version miRDeep2.0.0.7), miRPlant (version miRPlant_V5) and miReap (version mireap_0.2). The organisms we considered are *Danio rerio* (Cifuentes *et al.*, 2010), *Homo sapiens* (Shin *et al.*, 2010; Somel *et al.*, 2010; Vaz *et al.*, 2010; Hou *et al.*, 2011; Friedländer *et al.*, 2014; Kim *et al.*, 2016), *Mus musculus* (Bosson *et al.*, 2014; Groenendyk *et al.*, 2014; Noh *et al.*, 2014; Modzelewski *et al.*, 2015; Meng *et al.*, 2015), *Caenorhabditis elegans* (Garcia-Segura *et al.*, 2015), *Drosophila melanogaster* (Lee *et al.*, 2014), *Heliconius melpomene* (Surridge *et al.*, 2011), *Xenopus laevis* (Ahmed *et al.*, 2015) (animal datasets), *Solanum lycopersicum* (Lopez-Gomollon *et al.*, 2012; Kravchik *et al.*, 2014), *Glycine max* (Curtin *et al.*, 2016) and *Arabidopsis thaliana* (Wang *et al.*, 2011) (plant datasets). We have downloaded these datasets from the GEO (Barrett *et al.*, 2013) and SRA (Leinonen *et al.*, 2011) databases. We also generated an *A.thaliana* dataset, as described in the Supplementary File S2. A description of the data processing can be found in the Supplementary File S2. Information about the genomes used, accession numbers of small RNA datasets, trimmed adapter sequences and number of reads in each dataset can be found in the Supplementary File S4.

## 4 Results

To evaluate the performance of miRCat2, miRCat, miRDeep2, miRPlant and miReap, we generated the miRNA predictions, using all tools, on the same input datasets. We filtered the output of each tool as recommended by their authors (miRCat2: no filtering, miRCat: no filtering, miRDeep: filter by score cut-off of 0, miRPlant: filter by score cut-off of 4, miReap: no filtering). For each method and input set, we determined the average number of high-confidence and low-confidence miRNA precursors from miRBase v21 (Kozomara and Griffiths-Jones, 2014), the average number of new miRNA predictions, average specificity (percentage of miRBase annotated miRNAs within the output) and average sensitivity rates (percentage of miRNAs detected out of the total number of miRNAs expressed in the sample file). The averages for each organism are presented in Table 1 (full results for each dataset are described in the Supplementary File S5). We used miRBase as a reference of accepted/studied miRNAs, although we acknowledge its caveats (Saçar *et al.*, 2013).

**Table 1.** Performance comparison of benchmarked tools

Animals							Plants						
Organism	Tool	High-conf. miRNAs	Low-conf. miRNAs	Novel predictions	Specificity (%)	Sensitivity (%)	Organism	Tool	High-conf. miRNAs	Low-conf. miRNAs	Novel predictions	Specificity (%)	Sensitivity (%)
*H.sapiens*	miRCat2	159	83	72	78.6 (±9.1)	30.6 (±3.3)	*A.thaliana*	miRCat2	66	44	8	93.6 (±2.7)	38.3 (±2.7)
(23 datasets)	miRCat	122	67	27	87.9 (±5.8)	23.9 (±2.5)	(7 datasets)	miRCat	51	57	167	40.9 (±9)	37.9 (±1.8)
	miRDeep2	149	61	14	94 (±2.7)	26.5 (±4.5)		miRPlant	62	52	7	93.3 (±5.4)	39.3 (±14.9)
	miReap	148	108	227	52.3 (±14.3)	32.5 (±7.4)		miReap	6	8	121	14.5 (±8.5)	4.9 (±0.6)
*M.musculus*	miRCat2	147	25	23	90.5 (±7.5)	39.8 (±3.2)	*S.lycopersicum*	miRCat2	15	13	233	11.6 (±5)	44.2 (±12.8)
(21 datasets)	miRCat	124	20	20	88.5 (±8.3)	33.5 (±1.9)	(14 datasets)	miRCat	14	16	1204	2.7 (±1.1)	48 (±4.8)
	miRDeep2	117	14	2	98.6 (±2)	29.7 (±7.2)		miRPlant	11	7	45	30.3 (±7)	28.9 (±13.1)
	miReap	114	21	134	48.7 (±12.3)	31.6 (±8.5)		miReap	4	5	1619	0.7 (±0.3)	13.6 (±3.2)
*D.rerio*	miRCat2	141	145	42	93.6 (±2.4)	88.6 (±2.3)	*G.max*	miRCat2	N/A	129	269	32.7 (±3.8)	34.9 (±1.1)
(2 datasets)	miRCat	101	88	26	87.9 (±0.3)	58.2 (±2.5)	(2 datasets)	miRCat	N/A	149	865	15.4 (±4.5)	40.2 (±0.8)
	miRDeep2	120	111	27	89.7 (±1.3)	71.5 (±3.0)		miRPlant	N/A	80	74	52 (±0.7)	21.6 (±4.9)
	miReap	137	132	43	86.2 (±0.2)	82.9 (±0.2)		miReap	N/A	25	2243	1.2 (±0.3)	6.8 (±0.8)

*Note*: miRCat2 performs well consistently, with a good specificity and sensitivity trade-off, whereas miRCat and miReap struggle in terms of specificity, especially in plants. miRDeep2/miRPlant have good specificity, but lack in sensitivity.

To calculate the sensitivity, any miRNA precursor with at least one incident read was considered to be expressed in the given sample. This approach includes low abundance miRNAs, which may be difficult to predict, resulting in overall low sensitivity.

Comparing the prediction accuracy of miRCat2 with miRCat and miRDeep2/miRPlant, we observe that miRCat2 has comparable specificity to other methods, while achieving an improved sensitivity. In particular, we detect a higher number of known miRNAs, while avoiding the proportional increase in the number of new miRNAs predictions. For example, in *M.musculus*, miRCat2 detects 41 more miRNAs than miRDeep2, which has the highest specificity, while predicting only 21 additional (potentially new) miRNAs. Moreover, miRCat2 predicts the highest number of high-confidence miRBase miRNAs in all tests. For the *H.sapiens* samples, we see that miReap predicts more known miRNAs, although at a cost to specificity, as it generates a large number of new predictions (155 more than miRCat2), which may be false positives. In all other organisms miReap performs poorly, especially in plants, where both sensitivity and specificity are low.

To validate the miRCat2 predictions, we investigated whether the predicted mature miRNAs were dependent on Dicer/DCL1, Drosha and DGCR8 processing, known to be key factors in miRNA biogenesis in plants and animals, respectively. We expect bona fide miRNAs to have reduced expression in Dicer, Drosha, DGCR8 knock-out or knock-down versus wild-type samples. We consider a predicted miRNA as being downregulated in the mutant samples if the normalized expression is at least 2-fold lower in the mutant, when compared with the wild-type.

To evaluate the quality of the datasets, we produced sample versus sample scatter plots using the normalized expression levels in wild-type and mutant samples for miRBase miRNAs (see Supplementary File S1, Fig. S1.4). If the mutation was successful, we expect to see higher counts in the wild-type than in the mutant samples, therefore the plots should show a shift of the points above the diagonal; this pattern can be observed in the majority of cases. However, for *D.rerio* the pattern is not very clear; also in *G.max* and *M.musculus* the points are grouped on the diagonal. Nevertheless, in all cases more than a half of the points are situated above the diagonal. This suggests that these datasets contain overall lower percentages of differentially expressed miRNAs, and this is reflected in the cumulative plots too. Note that in the *H.sapiens* wild-type versus Drosha mutant, there are some miRNAs that are located below the diagonal (more highly expressed in the mutant). This is probably because they have a Drosha-independent biogenesis pathway and therefore appear to be more highly expressed in the mutant (Kim *et al.*, 2016).

**Fig. 2:**
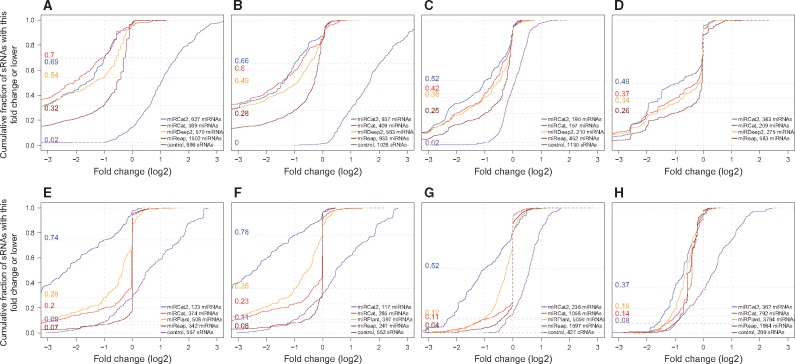
Cumulative plots of log_2_ fold changes of control versus mutant datasets, calculated on the output of miRCat2, miRCat, miRDeep2/miRPlant and miReap and a control dataset formed of tRNAs and snoRNAs. We present results for *H. sapiens* [subplots (**A**) Dicer and (**B**) Drosha knock-out], *M. musculus* [subplot (**C**)], *D. rerio* [subplot (**D**)], *A. thaliana* [subplots (**E**) and (**F**)], *S. lycopersicum* [subplot (**G**)] and *G. max* [subplot (**H**)]. miRCat2 has the highest percentage of DE miRNAs in all but one of the experiments, where it classifies as a close second to miRCat. (A) *Homo sapiens* wild-type versus Dicer knock-out. (B) *Homo sapiens* wild-type versus DROSHA knock-out. (C) *Mus musculus* wild-type versus DGCR8 knock-out. (D) *Danio rerio* wild-type versus Dicer knock-out. (E, F) *Arabidopsis thaliana* wild-type versus Dicer knock-down. (G) *Solanum lycopersicum* wild-type versus DCL1 knock-down. (H) *Glycine max* wild-type versus DCL1 knock-down

In the Supplementary File S1, Figure S1.3, we compare the performance of miRCat2, miRCat, miRDeep, miReap and miRPlant with and without filtering. For miRCat2, we used a score cut-off of 5 (empirically observed to separate most new predictions from conserved miRNAs). The filtering has some impact on both miRCat2 and miRDeep2 in *H.sapiens*. In plants however we observe that miRCat2 performs well irrespective of this filtering, with a particularly large impact for miRPlant. For comparability purposes, we computed the cumulative plots of log_2_ fold changes only on unfiltered outputs (see Fig. 2).

For tools with high prediction accuracy, we expect to see a significant differential expression (downregulation in the mutant samples) for the majority of the predicted miRNAs. As a control dataset containing reads independent in the miRNA biogenesis pathway, we use RFAM tRNA and snoRNA transcripts. As expected, their expression level is not decreased in the mutant samples; moreover, in the animal datasets the expression of these transcripts is upregulated, due to the stochasticity of the sequencing technology. In plant samples we observe little differential expression for the control sequences, as the biogenesis of plant sRNAs is more complex. All tools produce a substantially different cumulative differential expression curve compared to the control dataset; miRCat2 performs better than other tools in all but one of the experiments.

In the *H.sapiens* versus Dicer knock-out sample (see Fig. 2A), we observe that miRCat2 is a close second to miRCat, whereas in plant datasets there is a substantial gap between miRCat2 and the other tools, supporting the improved accuracy of miRCat2. For *S.lycopersicum*, miRCat2 shows a low specificity when detecting annotated miRNAs due to a low number of entries for this species (77 annotated precursors). However, the cumulative plots indicate that the new predictions are likely true miRNAs which have not yet been annotated in *S.lycopersicum* (54 out of the 190 new predictions are orthologs of plant miRNAs; see Supplementary File S6 for details).

Next, we produced cumulative plots on the differential expression frequency only for the sequences that were not previously included in miRBase and therefore are potential new miRNAs (see Supplementary File S1, Fig. S1.5). This subset contains a high proportion of putative miRNAs downregulated in the mutant samples, although to a lesser extent than the sequences included in the plots for all predictions. We observe no change in the ranking of the tools, miRCat2 performing better than the other tools in each of the experiments. In *M.musculus* we observe a decrease in the percentage of sequences with at least a 2-fold change in all tools, due to the low number of new predictions. The high percentage of differentially expressed sequences among new predictions, especially in plants, indicates that these sequences are likely to be bona fide miRNAs.

To evaluate the low overall sensitivity rates, we created cumulative plots using as input the miRNAs present in the datasets, but not detected by each tool. We expect these annotated miRNAs to have low counts in the input samples. Some of these sequences could also be misannotations in miRBase and exhibit features not consistent with canonical miRNA structure and biogenesis. As a result, their expression would not be affected in the mutant samples. Consequently, we expect to see a smaller differential expression between the wild-type and mutant samples in the cumulative plot, i.e. a curve closer to the control line. In the Supplementary File S1, Figure S1.6, we observe a clear change in the shape of the cumulative plots for each tool (especially for miRCat2), suggesting that these miRNAs might not present the canonical miRNA features or were lowly expressed in the datasets analyzed. Also, it is notable that miRCat2 consistently performs well, suggesting that it is less prone to false positives than other methods.

All miRCat2 new predictions are given in the Supplementary File S7.

## 5 Discussion

We presented a new tool for miRNA prediction, miRCat2, applicable on both plant and animal data, which can be run both from the UEA small RNA Workbench graphical interface and from the command line.

We tested miRCat2 on 10 model organisms and compared its results with four commonly used tools for miRNA discovery (miRCat, miRDeep2, miRPlant and miReap). miRCat2 shows a good trade-off between sensitivity and specificity (relative to miRBase annotation), performing well in both metrics, whereas other tools generally performed well only for one of these measures. More specifically, miRDeep2 and miRPlant had good specificity rates, but lacked in sensitivity (annotated miRNAs are not predicted). miReap had a good sensitivity in animals, but lacked in specificity, allowing a high number of new predictions, which could potentially contain false positives.

To evaluate the accuracy of the predictions, we used the miRBase annotations and the objective and biologically meaningful mutant test (using Dicer/DCL1, Drosha and DGCR8 mutants). This approach alleviated the lack of in-depth miRNA annotations for some model organisms (Saçar *et al.*, 2013). We have shown using the comparison of wild-type and mutant datasets, in the cumulative plots, that miRCat2 generally performs better than all other tools tested, both overall and when confirming novel annotations. The tool also remains consistent in its predictions across all animal and plant data, whereas the other tools tend to perform better only on some of the organisms: miRCat and miRDeep2 perform well in *H.sapiens* and *D.rerio*, whereas miRPlant performs well in *A.thaliana*.

miRCat2 is based on a new peak selection and feature-filtering algorithm, i.e. it can only detect miRNAs with conservative secondary structures and miRNA-specific features. In animals, the pre-miRNAs have a well-defined structure with little fluctuations, making the detection of miRNAs easier. In plants, however, there is a higher degree of variability in miRNA hairpin length (Cuperus *et al.*, 2011) and hairpins can contain multiple loops and additional smaller hairpins (Chen, 2005; Xie *et al.*, 2015). These features make the plant miRNA detection challenging. Therefore, rule-based tools, such as miRCat2, miRCat, miRDeep2, miRPlant and miReap, may perform poorly on plant data, missing miRNAs with uncharacteristic features or allowing a large number of false positives. The results for plant data show that miReap performs poorly, displaying low sensitivity and specificity and also the poorest performance in the comparison with mutant datasets. This indicates high false positive and false negative rates and, although it performs better on animal data, miReap should probably not be used for plant miRNA prediction.

Another criterion that influences the outcome of miRCat2 is the read abundance of an miRNA locus: miRCat2 may miss miRNAs that are lowly expressed in the input samples due to the calculations used to test against an RUD, for the identification of peaks. Nevertheless, the detection of low abundance miRNAs is a common issue for all miRNA prediction tools. This is not necessarily a disadvantage, as low read counts would suggest that the miRNA may not be expressed in that particular sample. In another sample where the miRNA is more highly expressed it is more likely that it would be predicted. miRCat2 generates a score as a mean of ranking its predictions and performs well irrespective of a filtering based on this score. This suggests that the core algorithm is robust.

In terms of run time, miRCat2 compares favorably with miRDeep2, although miReap was faster. For example, on a *H.sapiens* dataset, containing approximately 34.5 million reads, miRCat2 generated the results in 3h50m, whereas miRDeep2 generated the results in 5h15m (all tests performed on a Linux server with CentOS 5.11 operating system, 144 GB of memory and 2 Intel Xeon X5550 processors). In terms of memory usage, the amount allocated for one miRCat2 run is user-defined making it versatile to run on a wide range of specifications.

In conclusion, miRCat2 provides improved identification and characterization of new miRNAs over a range of organisms that are not predicted by other tools. It should therefore contribute to a better, more in-depth understanding of miRNAs, both in plants and animals.

## Funding

This work was supported by Biotechnology and Biological Sciences Research Council (BBSRC) [BB/L021269/1 to V.M., M.S. and T.D.].

*Conflict of Interest*: none declared.

## Supplementary Material

Supplementary Data SD1

Supplementary Data SD2

Supplementary Data SD3

Supplementary Data SD4

Supplementary Data SD5

Supplementary Data SD6

Supplementary Data SD7
