# Supplementary material for: miRCat2: accurate prediction of plant and animal microRNAs from next-generation sequencing datasets
Source: Bioinformatics. 2017 Apr 12;33(16):2446–54. doi: 10.1093/bioinformatics/btx210 (PMC5870699; doi:10.1093/bioinformatics/btx210)
Supplement: Supplementary Data SD1 [file supplementary_data_sd1_btx210.pdf]

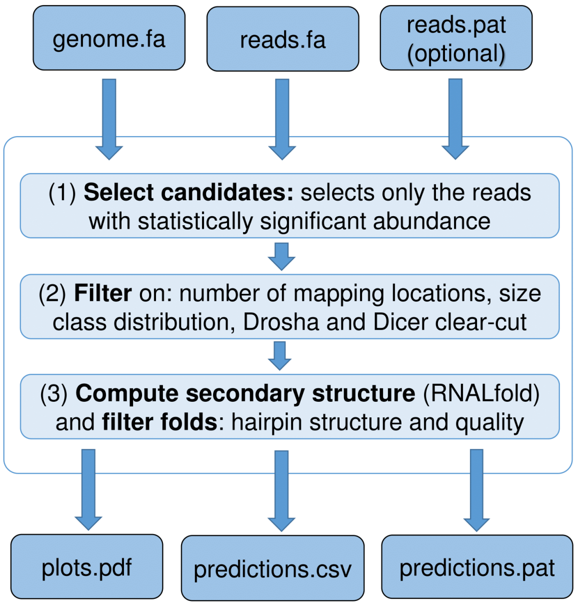

Fig. SD1.1. Workflow of the miRCat2 algorithm. The inner light-blue boxes represent processes, the outer dark-blue boxes are input and output files. The file formats are: .fa, fasta; .pat, PatMaN output; .csv, csv spreadsheet. A detailed description of these steps can be found in the methods section.

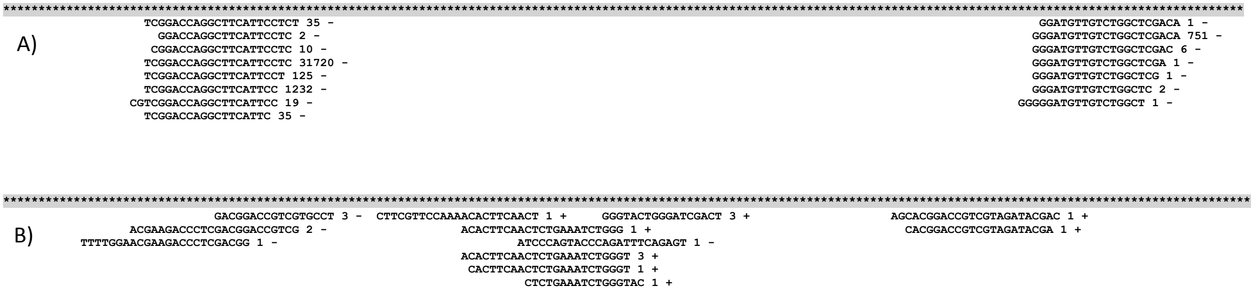

Fig. SD1.2. Distribution of reads for a known miRNA locus A) and a random locus on the genome with incident degradation reads B). For each incident read we present, on the right, its abundance (read count),and the matching strand (+/-). A) Distribution of reads for sly-MIR166c (S. lycopersicum), on chromosome 1, positions 84381885 - 84382061. This shows the expected miRNA locus pattern, with a characteristic two-peak alignment corresponding to the 5'/3' miRNAs. B) Random distribution of reads for S. lycopersicum, on chromosome 1, positions 2076029 - 2076206. The lack of location, size class or abundance specificity, corroborated with the lack of a hairpin-like secondary structure, indicates that this alignment doesn't correspond to a miRNA locus.

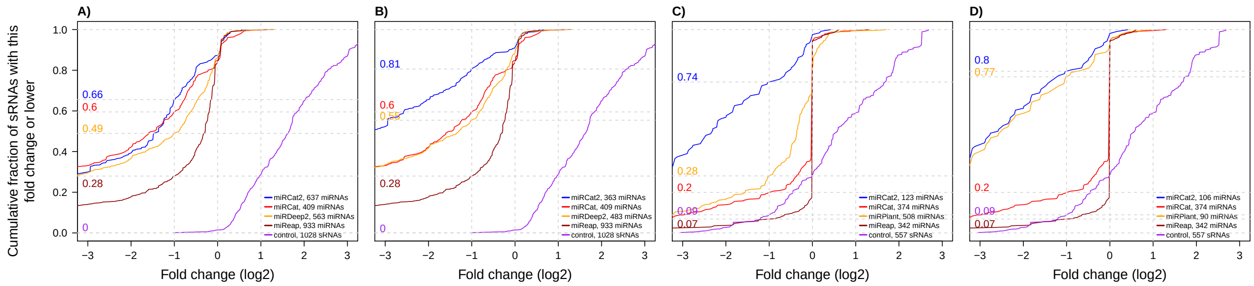

Fig. SD1.3. Comparison of filtered vs not filtered results for H. sapiens (subplots A) and B)) and A. thaliana (subplots C) and D)) data. In each plot we represent the cumulative distribution of differential expression for predictions conducted with miRCat2, miRCat, miRDeep2/miRPlant and miReap. The results were filtered based on the recommended cut-off of the score for miRDeep2 (0) and miRPlant (4) and a value of 5 for miRCat2, empirically determined. We observe that for both plant and animal data, the filtering has an effect on the performance of the tools. A) H. sapiens wildtype vs. DROSHA knock-out, before filtering. B) H. sapiens wildtype vs. DROSHA knock-out, after filtering. C) A. thaliana wildtype vs. DCL1 knock-down, before filtering. D) A. thaliana wildtype vs. DCL1 knock-down, after filtering.

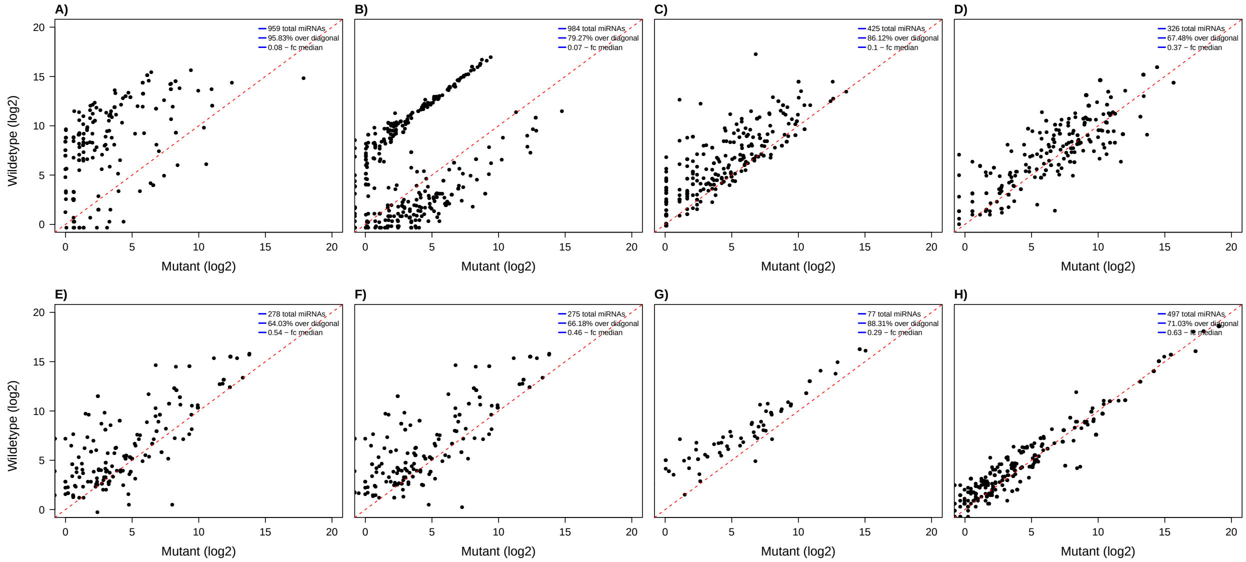

Fig. SD1.4. Correlation plots of normalized average abundances for expressed miRBase miRNAs in the wildtype, compared to mutant samples. We present results for H. sapiens (subplots (A) Dicer and (B) Drosha knock-out), M. musculus (subplot (C)), D. rerio (subplot (D)), A. thaliana (subplots (E) and (F)), S. lycopersicum (subplot (G)) and G. max (subplot (H)). The plots give information about the percentage of miRNAs that are more abundant in the wildtype (above diagonal) and the median fold change, where a fold change of 0.5 means the sequence is down-regulated in the mutant. (A) H. sapiens wildtype vs. Dicer knock-out. (B) H. sapiens wildtype vs. DROSHA knock-out. (C) M. musculus wildtype vs. DGCR8 knock-out. (D) D. rerio wildtype vs. Dicer knock-out. (E-F) A. thaliana wildtype vs. Dicer knock-down. (G) S. lycopersicum wildtype vs. DCL1 knock-down. (H) G. max wildtype vs. DCL1 knock-down.

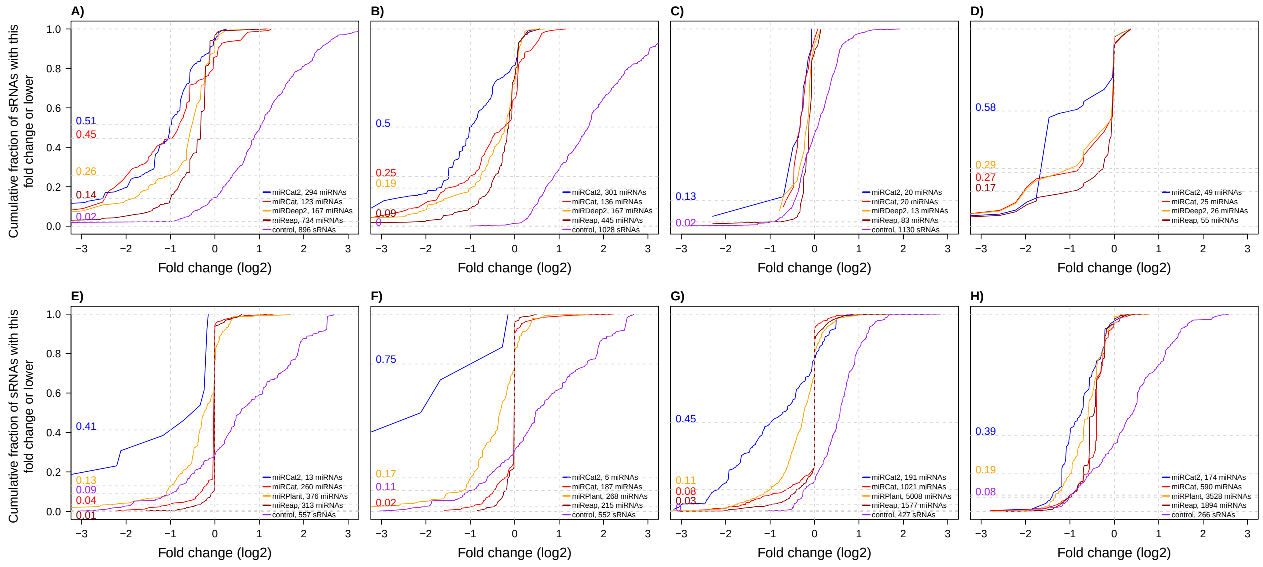

Fig. SD1.5. Cumulative plots of log_2 fold changes of control vs. mutant datasets, calculated on the new predictions of miRCat2, miRCat, miRDeep2/miRPlant and miReap and a control dataset formed of tRNAs and snoRNAs. We present results for H. sapiens (subplots A) Dicer and B) Drosha knock-out), M. musculus (subplot C)), D. rerio (subplot D)), A. thaliana (subplots E) and F)), S. lycopersicum (subplot G)) and G. max (subplot H)). miRCat2 has the highest percentage of DE miRNAs in all of the experiments. A) H. sapiens wildtype vs. Dicer knock-out. B) H. sapiens wildtype vs. DROSHA knock-out. C) M. musculus wildtype vs. DGCR8 knock-out. D) D. rerio wildtype vs. Dicer knock-out. (E-F) A. thaliana wildtype vs. Dicer knock-down. G) S. lycopersicum wildtype vs. DCL1 knock-down. H) G. max wildtype vs. DCL1 knock-down.

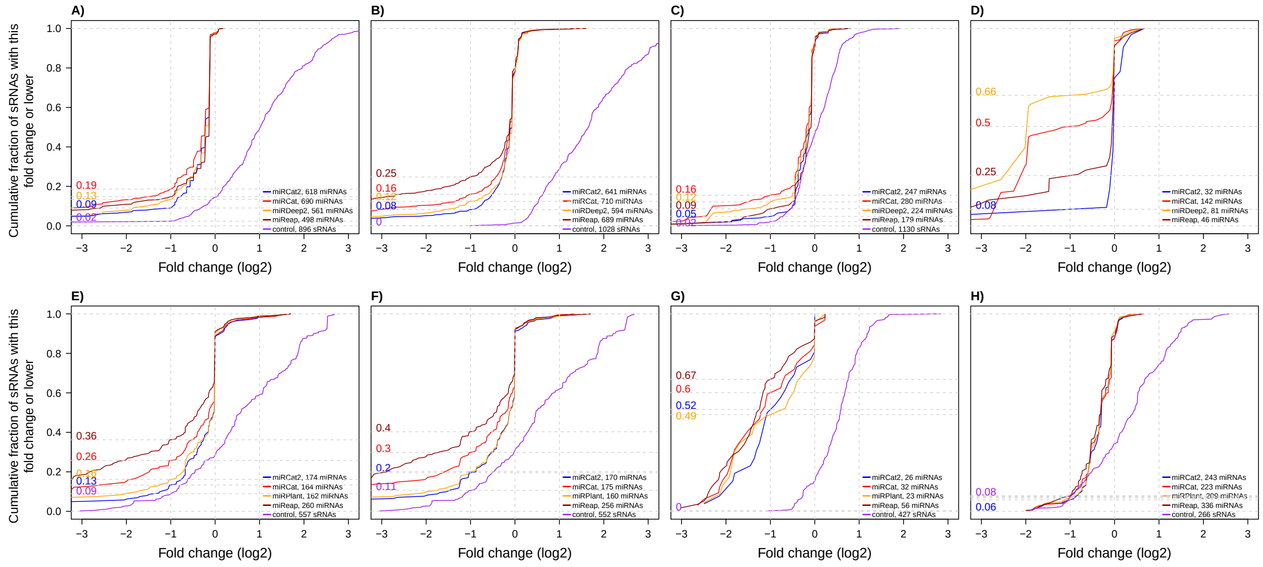

Fig. SD1.6. Cumulative plots of log_2 fold changes of control vs. mutant datasets, calculated on miRBase miRNAs present in the datasets, but not detected by the predictions of miRCat2, miRCat, miRDeep2/miRPlant and miReap and on a control dataset formed of tRNAs and snoRNAs. We present results for H. sapiens (subplots (A) Dicer and (B) Drosha knock-out), M. musculus (subplot (C)), D. rerio (subplot (D)), A. thaliana (subplots (E) and (F)), S. lycopersicum (subplot (G)) and G. max (subplot (H)). We expect to see a smaller differential expression between the wildtype and mutant samples in the cumulative plot i.e. a curve closer to the control line. miRCat2 presents the lowest differential expression in all experiments, suggesting that it is less prone to false positives than other methods. (A) H. sapiens wildtype vs. Dicer knock-out. (B) H. sapiens wildtype vs. DROSHA knock-out. (C) M. musculus wildtype vs. DGCR8 knock-out. (D) D. rerio wildtype vs. Dicer knock-out. (E-F) A. thaliana wildtype vs. Dicer knock-down. (G) S. lycopersicum wildtype vs. DCL1 knock-down. (H) G. max wildtype vs. DCL1 knock-down.
