# Supplementary material for: miRCat2: accurate prediction of plant and animal microRNAs from next-generation sequencing datasets
Source: Bioinformatics. 2017 Apr 12;33(16):2446–54. doi: 10.1093/bioinformatics/btx210 (PMC5870699; doi:10.1093/bioinformatics/btx210)
Supplement: Supplementary Data SD2 [file supplementary_data_sd2_btx210.pdf]

1 miRCat2 clustering algorithm

This algorithm is part of the step for filtering of the sequences, that checks whether the candidates have a miRNA-like alignment of incident reads. It is used to determine if the selected sequences present evidence of precise processing of the pre-miRNA by Drosha (animals) and Dicer (plants and animals) (Kim, 2005; Bartel, 2004; Chen, 2005) i.e. the presence of one or two peaks corresponding to the miRNA/miRNA*. This filtering step ensures that the majority of reads aligned to the miRNA/miRNA* location have a high overlap (are variants of each other), and have the same genomic orientation (Kim, 2005; Bartel, 2004; Chen, 2005).

We define a cluster as all sequences that map to the same genomic location, having the start and the end of the mapping position within $clear\_cut$ nts of each other. We chose $clear\_cut$ to be 3 nts, because during the miRNAs biogenesis, many isomiRs are also generated within these bounds (sequences that have variations of a few nts with respect to the reference miRNA sequence) (Morin *et al.*, 2008).

The rules for an sRNA sequence s (with sRNA beginning position b_s and ending position e_s) to be considered to belong to a cluster C (with cluster beginning position b_C and ending position e_C) are:

- if $b_s \geq b_C$ and $e_s \leq e_C$, then the sRNA location is completely inside the cluster boundaries, therefore $s \in C$;
- if $b_C \geq b_s$ and $e_C \leq e_s$, then the sRNA location is completely covering the cluster location, therefore $s \in C$;
- if ($b_s - b_C \leq clear\_cut$ and $b_s - b_C \geq 0$) or ($e_s - e_C \leq clear\_cut$ and $e_s - e_C \geq 0$), then the sRNA location is with $clear\_cut$ nts to the right or left from the cluster location, therefore $s \in C$.
- we define mid_s to be $mid_s = \frac{(e_s - b_s)}{2} + b_s$ and mid_C to be $mid_C = \frac{(e_C - b_C)}{2} + b_C$. If $|mid_C - mid_s| \leq clear\_cut$, then the middle of the sRNA location is with $clear\_cut$ nts to the right or left from the middle of the cluster location, therefore $s \in C$;
- otherwise $s \notin C$.

2 Fold change computation

To validate miRNA predictions, we estimate fold changes between wild type and mutants in the miRNA biogenesis pathway. To do this, we consider only the genome mapping reads. To compare datasets with different sequencing depths, we normalize all abundances using the reads per million (RPM) method (Mortazavi *et al.*, 2008) to the median total count (MTC) of each experiment (McCormick *et al.*, 2011; Dillies *et al.*, 2013). Briefly, we sum the abundances of genome mapping reads in each sample to obtain the total for each library; next we calculate the median of total counts for each comparison (the MTC value). We normalize the abundance of each read using: $normalized\_count = \frac{count}{total\_library\_count} * MTC$ (McCormick *et al.*, 2011).

To create a control dataset, as suggested in (Friedländer *et al.*, 2014), containing reads whose abundances are unlikely to be affected by the mutations on the miRNA pathway, the reads in each experiment were mapped to a file containing tRNAs and snoRNAs of the respective species, using PatMaN, full length, with 0 gaps and 0 mismatches. The control file was created downloading tRNAs and snoRNAs from the RFAM database (Nawrocki *et al.*, 2014) through the RNAcentral web service (RNAcentral Consortium, 2014) (http://rnacentral.org/). For each RFAM transcript, we compute its abundance as the algebraic sum of the normalized abundances of mapped reads, for each condition.

We then calculate the log_2 fold change using the normalized abundances of all predicted miRNAs, from each tool. The log_2 fold change for each miRNA is calculated for each set of replicates as the ratio between the median value of normalized abundances from the mutant samples to the median value of normalized abundances from the wildtype (control) samples. We use an offset approach, adding a count of 10 to both numerator and denominator, to avoid divisions by zero and cases where lowly expressed sequences appear to be differentially expressed (Mohorianu *et al.*, 2011). Next, we compare the percentage of reads that are significantly down regulated in the mutant samples ($\geq$ 2-fold downregulated).

3 Data processing

All samples downloaded from GEO (Barrett *et al.*, 2013) or SRA (Leinonen *et al.*, 2010) databases were processed as follows: files were transformed to fasta format (using the sratoolkit-2.4.2 (Leinonen *et al.*, 2010)). 3' adapters were trimmed and sequences longer than 16 nt were kept for the subsequent steps. All *A. thaliana* samples that we sequenced were transformed from fastq to fasta format, then the 3' adapters and the HD tags were trimmed (using the UEA small RNA Workbench); sequences longer than 16 nt were kept for the subsequent steps. Next, all files were collapsed into non-redundant format (for each sRNA we kept the sequence and its abundance). Then the files were aligned full length, with 0 gaps and 0 mismatches to the respective reference genome using PatMaN (Prüfer *et al.*, 2008). For miRDeep2, the reads were mapped using mapper.pl; to make the results comparable with the other methods used, the sequences which mapped with mismatches were discarded. All software was run on the processed datasets with their default parameters.

4 *A. thaliana* wildtype and DCL1 mutant small RNA sequencing

4.1 Plant materials

Seeds of *Arabidopsis thaliana* cv. Columbia *dcl1-7* (*sin1-1*) mutants from NASC The European Arabidopsis Stock Centre (stock ID N6953) were stratified at 4°C for 48h and germinated under long-day conditions with 16h daytime at 22°C and 8h night time at 18°C. The plants were grown under the same conditions. Leaves were harvested and genomic DNA were extracted as previously described (Amani *et al.*, 2011) for genotyping. Heterozygous individuals were identified through PCR-sequencing for seed increase.

The seeds from wild type and heterozygous individuals were germinated and the plants were grown under the same conditions as above. The homozygous plants were identified by the phenotypes (Lang *et al.*, 1994). When the flower buds in both wild type and mutant plants were visible but without opening flowers, the leaves of nine plants were harvested and stored at -80°C for three biological replicates. One replicate consists of a pool of leaves from three individual plants.

4.2 Genotyping analysis

Dcl-1 specific primers: 5'-TGTCACCACGCTGTCAAGAA-3' (forward) and 5'-GCAGCTTTGTCATACTCGACG-3'(reverse) were used for PCR. The final concentrations for the reaction components are: 1X GoTaq® Reaction Buffer, 0.2mM dNTP each, 0.5uM primer each, 1.25U GoTaq® DNA Polymerase and 6ng/uL genomic DNA. PCR was performed with the following programme: 2min at 95°C, 35 cycles of 30 sec at 95°C, 30 sec at 60°C and 45sec at 72°C, and a final extension for 5min at 72°C. The PCR products were purified using the Zymoclean™ Gel DNA Recovery Kit following the manufacturer's protocol, and sequenced. Heterozygous samples contain both wild type and mutant sequences.

4.3 RNA extraction and small RNA library construction

Total RNA of both wild type and mutant leaf tissue was extracted using Tri-Reagent (Ambion) following manufacturer's protocol. Small RNA (sRNA) of each sample was further enriched using the mirVana miRNA isolation kitTM (Ambion) according to the manufacturer's instructions. The sRNA libraries were constructed using 1 μg of enriched sRNA fraction based on the previously published protocol by Xu et al., 2015 (Xu *et al.*, 2015).

The libraries were barcoded, pooled and sequenced on a single HiSeq2500 lane. The raw fastq files and processed csv files are publicly available on Gene Expression Omnibus (GEO) (Barrett *et al.*, 2013) under accession number GSE90771 (GSM2412286 to GSM2412288 are the wild type samples and GSM2412289 to GSM2412291 are the DCL1 mutant samples).

References

Amani,J., Kazemi,R., Abbasi,A.R. and Salmanian,A.H. (2011) A simple and rapid leaf genomic DNA extraction method for polymerase chain reaction analysis. *Iranian journal of biotechnology,* **9** (1), 69–71.

Barrett,T., Wilhite,S.E., Ledoux,P., Evangelista,C., Kim,I.F., Tomashevsky,M., Marshall,K.A., Phillippy,K.H., Sherman,P.M., Holko,M. *et al.* (2013) NCBI GEO: archive for functional genomics data sets - update. *Nucleic acids research,* **41** (D1), D991–D995.

Bartel,D.P. (2004) MicroRNAs: genomics, biogenesis, mechanism, and function. *cell,* **116** (2), 281–297.

Chen,X. (2005) MicroRNA biogenesis and function in plants. *FEBS letters,* **579** (26), 5923–5931.

Dillies,M.A., Rau,A., Aubert,J., Hennequet-Antier,C., Jeanmougin,M., Servant,N., Keime,C., Marot,G., Castel,D., Estelle,J. *et al.* (2013) A comprehensive evaluation of normalization methods for Illumina high-throughput RNA sequencing data analysis. *Briefings in bioinformatics,* **14** (6), 671–683.

Friedländer,M.R., Lizano,E., Houben,A.J., Bezdan,D., Báñez-Coronel,M., Kudla,G., Mateu-Huertas,E., Kagerbauer,B., González,J., Chen,K.C. *et al.* (2014) Evidence for the biogenesis of more than 1,000 novel human microRNAs. *Genome Biol,* **15** (4), R57.

Kim,V.N. (2005) MicroRNA biogenesis: coordinated cropping and dicing. *Nature reviews Molecular cell biology,* **6** (5), 376–385.

Lang,J.D., Ray,S. and Ray,A. (1994) sin 1, a mutation affecting female fertility in Arabidopsis, interacts with mod 1, its recessive modifier. *Genetics,* **137** (4), 1101–1110.

Leinonen,R., Sugawara,H. and Shumway,M. (2010) The sequence read archive. *Nucleic acids research,* , gkq1019.

McCormick,K.P., Willmann,M.R. and Meyers,B.C. (2011) Experimental design, preprocessing, normalization and differential expression analysis of small RNA sequencing experiments. *Silence,* **2** (1), 1.

Mohorianu,I., Schwach,F., Jing,R., Lopez-Gomollon,S., Moxon,S., Szittya,G., Sorefan,K., Moulton,V. and Dalmay,T. (2011) Profiling of short RNAs during fleshy fruit development reveals stage-specific sRNAome expression patterns. *The Plant Journal,* **67** (2), 232–246.

Morin,R.D., O'Connor,M.D., Griffith,M., Kuchenbauer,F., Delaney,A., Prabhu,A.L., Zhao,Y., McDonald,H., Zeng,T., Hirst,M. *et al.* (2008) Application of massively parallel sequencing to microRNA profiling and discovery in human embryonic stem cells. *Genome research,* **18** (4), 610–621.

Mortazavi,A., Williams,B.A., McCue,K., Schaeffer,L. and Wold,B. (2008) Mapping and quantifying mammalian transcriptomes by RNA-Seq. *Nature methods,* **5** (7), 621–628.

Nawrocki,E.P., Burge,S.W., Bateman,A., Daub,J., Eberhardt,R.Y., Eddy,S.R., Floden,E.W., Gardner,P.P., Jones,T.A., Tate,J. *et al.* (2014) Rfam 12.0: updates to the RNA families database. *Nucleic acids research,* , gku1063.

Prüfer,K., Stenzel,U., Dannemann,M., Green,R.E., Lachmann,M. and Kelso,J. (2008) PatMaN: rapid alignment of short sequences to large databases. *Bioinformatics,* **24** (13), 1530–1531.

RNAcentral Consortium (2014) RNAcentral: an international database of ncRNA sequences. *Nucleic acids research,* , gku991.

Xu,P., Billmeier,M., Mohorianu,I., Green,D., Fraser,W.D. and Dalmay,T. (2015) An improved protocol for small RNA library construction using high definition adapters. *Methods Next Generation Seq,* **2**, 1–10.
