# Supplementary material for: miRCat2: accurate prediction of plant and animal microRNAs from next-generation sequencing datasets
Source: Bioinformatics. 2017 Apr 12;33(16):2446–54. doi: 10.1093/bioinformatics/btx210 (PMC5870699; doi:10.1093/bioinformatics/btx210)
Supplement: Supplementary Data SD3 [file supplementary_data_sd3_btx210.pdf]

1 Prameters of miRCat2

**User-configurable parameters**				
**Parameter name**	**Description**	**Default value animals**	**Default value plants**	**Justification for value**
min_len	Minimum length of a miRNA	20	20	Includes sequences that fall out of the regular miRNA size class
max_len	Maximum length of a miRNA	24	23	Includes sequences that fall out of the regular miRNA size class
min_fold_len	Minimum length of a hairpin	40	45	Lower value than minimum fold length for most organisms
max_fold_len	Maximum length of a hairpin	100	250	Higher value than maximum fold length for most organisms
max_amfe	Maximum value for the AMFE for a miRNA precursor	-22	-22	Empirically determined
complex	Complexity of sequence	0.90	0.90	Empirically determined
gaps_miRNA	Maximum number of consecutive gaps on the hairpin on the miRNA location	4	4	Empirically determined
repeats	Maximum number of times a sRNA can map to the genome (usually miRNAs map to a limited number of locations)	25	25	a miRNA sequence does not map repeated times to the reference genome
pVal	Threshold for the RANDfold output value	0.05	0.05	Statistically significant value
complex_loop	If a hairpin with multiple loops between the miRNA and miRNA* is allowed	false	true	Complex secondary structures have been previously seen in plants, but never in animals. If a complex loop is permitted, it should not contain more than 3 loops.
no_loop	Maximum number of bulks in the loop area of the precursor	0	3	Empirically determined
clear_cut_percent	Percent of incident reads that should fall between the same start and end positions as the miRNA	0.95	0.92	Empirically determined, plant data is more variable
RANDfold	If RANDfold should be computed	false	false	Results are accurate without it and it slows the algorithm. Recomended if wanting to further restrict the results

Table SD2.1. Parameters involved in the algorithm of miRCat2, that are user-configurable. The parameters are presented with their default values and the justification for using the respective value, for both animal and plant data.

Predefined parameters (user cannot change)				
**Parameter name**	**Description**	**Default value animals**	**Default value plants**	**Justification for value**
min_orientation	Percent of the reads that have the same strand on the hairpin	0.8	0.8	Empirically determined
min_pared_perc	Minimum percentage of nts that should be paired on a hairpin	0.5	0.5	Empirically determined
min_pared_nucl	Minimum number of nts that should be paired on a hairpin	15	15	Empirically determined
overlap_percent	Maximum percent of sRNAs in an adjacent cluster overlapping with the miRNA cluster in order to be considered a clear cut	0.05	0.05	Empirically determined
fuzzy	Percent of all reads aligned to the hairpin that should map in accordance to Dicer/DCL1 and Drosha products	0.9	0.9	Empirically determined
min_fold_len	Minimum length of a hairpin	40	45	Minimum length of the miRNA, miRNA* and loop added together
window	Number of nts a split of the genome has.	300	500	Large enough to contain a miRNA hairpin but small enough to represent a significant local context of reads
subwindow	Number of nts a split of the window has.	20	20	It can cover at least a half of the longer miRNAs (25nts), but not more than then maximum length
window_overlap	Number of nts two adjacent windows overlap	100	100	ensures that adjacent windows are not isolated, but they influence each other
depth	Number of iterations to perform the KLD on genome location if removing a sRNA does not bring it closer to a RUD	4	4	Empirically determined
rud_val	Threshold for the KLD below which we consider the distribution to be a RUD	1.23	1.23	Empirically determined
min_loop	Minimum number of nts that the loop should have	3	3	Empirically determined
clear_cut	Number of nts a sRNA can be shifted regarded to a miRNA in order to be considered to have the same start/end (isomir)	3	3	Empirically determined
under_clear_cut	Percent of sRNA in a cluster with the same cut in order to be considered a clear cut. This is considered if the clear_cut_percent fails on one of the sides of the miRNA	0.7	0.7	Empirically determined
min_size	Minimum length of a sRNA in the file	16	16	Sequences smaller than 16 nt are adapter-adapter sequences and should not exist in the dataset
max_size	Maximum length of a sRNA in the file	40	40	There are usually very few sequences with length over 40 in a sRNA dataset
offset	Value added to the reads distribution in order to avoid division by 0	1	1	Minimum read abundance
offset_low	Value added to the reads distribution in order to avoid division by 0 when read abundance is low	offset*0.9	offset*0.9	Offset becomes more significant than actual read abundances
plateau_range	Number of subwindow to be included in the local peak detection on both sides of the miRNA candidate	4	6	Empirically determined
3‘overhang	Number of nts the miRNA* is shifted compared to the miRNA	2	2	miRNA biogenesis

Table SD2.2. Predefined parameters involved in the algorithm of miRCat2, that cannot be changed by the user. The parameters are presented with their default values and the justification for using the respective value, for both animal and plant data.
